# Older Adults’ Engagement and Mood During Robot-Assisted Group Activities in Nursing Homes: Development and Observational Pilot Study

**DOI:** 10.2196/48031

**Published:** 2023-12-25

**Authors:** Alexandra Tanner, Andreas Urech, Hartmut Schulze, Tanja Manser

**Affiliations:** 1 School of Applied Psychology University of Applied Sciences and Arts Northwestern Switzerland Olten Switzerland; 2 City of Bern (Digital Stadt Bern) Bern Switzerland

**Keywords:** human-robot interaction, social robot, nursing home, observational research, group activity, observational, pilot study, robot, engagement, mood, well-being, cognitive, elderly, social robot, nursing, aging

## Abstract

**Background:**

Promoting the well-being of older adults in an aging society requires new solutions. One resource might be the use of social robots for group activities that promote physical and cognitive stimulation. Engaging in a robot-assisted group activity may help in the slowdown of physical and cognitive decline in older adults. Currently, our knowledge is limited on whether older adults engage in group activities with humanlike social robots and whether they experience a positive affect while doing so. Both are necessary preconditions to achieve the intended effects of a group activity.

**Objective:**

Our pilot study has 2 aims. First, we aimed to develop and pilot an observational coding scheme for robot-assisted group activities because self-report data on engagement and mood of nursing home residents are often difficult to obtain, and the existing observation instruments do have limitations. Second, we aimed to investigate older adults’ engagement and mood during robot-assisted group activities in 4 different nursing care homes in the German-speaking part of Switzerland.

**Methods:**

We developed an observation system, inspired by existing tools, for a structured observation of engagement and mood of older adults during a robot-assisted group activity. In this study, 85 older adult residents from 4 different care homes in Switzerland participated in 5 robot-assisted group activity sessions, and they were observed using our developed system. The data were collected in the form of video clips that were assessed by 2 raters regarding engagement (direction of gaze, posture as well as body expression, and activity) and mood (positive and negative affects). Both variables were rated on a 5-point rating scale.

**Results:**

Our pilot study findings show that the engagement and mood of older adults can be assessed reliably by using the proposed observational coding scheme. Most participants actively engaged in robot-assisted group activities (mean 4.19, SD 0.47; median 4.0). The variables used to measure engagement were direction of gaze (mean 4.65, SD 0.49; median 5.0), posture and body expression (mean 4.03, SD 0.71; median 4.0), and activity (mean 3.90, SD 0.65; median 4.0). Further, we observed mainly positive affects in this group. Almost no negative affect was observed (mean 1.13, SD 0.20; median 1.0), while the positive affect (mean 3.22, SD 0.55; median 3.2) was high.

**Conclusions:**

The developed observational coding system can be used and further developed in future studies on robot-assisted group activities in the nursing home context and potentially in other settings. Additionally, our pilot study indicates that cognitive and physical stimulation of older adults can be promoted by social robots in a group setting. This finding encourages future technological development and improvement of social robots and points to the potential of observational research to systematically evaluate such developments.

## Introduction

### Background

Given the global phenomenon of aging populations, strategies to reduce the risk of physical and cognitive decline and the associated consequences on the well-being of older adults and their ability to cope with everyday life are urgently needed [1]. One resource in this context might be the use of the so-called social robots. According to Anzalone and colleagues [2], social robots can be understood as “machines that humans should perceive as realistic, effective partners, able to communicate and cooperate with them as naturally as possible interestingly enough.” The acceptance of social robots and their potential to promote the well-being of older adults have been explored and demonstrated in several studies [3-8]. Most of the robots studied are animallike, with PARO [9], a seal-shaped robot, being a prominent example [2-5,10,11]. However, animallike companion robots are not multifunctional and their interactions are not sufficient for those who require care and support. A study comparing animallike and humanlike social robots in group settings provided the first evidence that humanlike robots have greater effects on cognitive training than animallike robots [12], which brings humanlike social robots into the focus of research for group activities for older adults. This so-called third generation of social robots, including Nao, Pepper, QT, Sophia, Jack, LOVOT, or Tessa [12-18], continue to evolve, as new software is developed and released into the market [13]. Their humanlike forms [19] and integrated voice capability allow for interactions through facial expression, gestures, and voice. Thus, these robots can support cognitive and physically stimulating exercises, which in combination, achieve the best results in maintaining cognitive abilities in older adults [1].

Few studies [7,12,13,20-25] have investigated whether older adults actively engage in and experience positive moods during these activities. Since mood and engagement are crucial for the effectiveness of such group activities with a humanlike social robot, this study aims to explore these 2 constructs empirically. In doing so, we chose the method of systematic behavioral observation, because self-report data of older adults in nursing homes are often difficult to obtain and might interfere with their experience of the activity itself [9]. As no suitable observational coding scheme could be identified in the literature, a second aim of this study was the development and piloting of an observational coding scheme. In summary, this pilot study addresses the following questions: (1) can the engagement and mood of older adults in a robot-assisted group activity be assessed through systematic behavioral observation? and (2) do older adults actively engage in a robot-assisted group activity and what mood (ie, positive or negative affect) can be observed in the group during such a robot-assisted group activity?

### Related Work

A review identified group activities for older adults assisted by social robots in 5 domains: affective therapy, cognitive training, social facilitation, companionship, and physiological therapy [7]. Three studies [12,13,20] showed a great potential of humanlike robots in group activities, with broader functionalities for physical activities. The first indications that older adults liked to participate in robot-assisted group activities for physical activities are shown in [21,22]. The robot NAO was found suitable to be used in group settings for moving, memory training, entertainment, music, dancing, and games [23]. One study showed that older adults in a nursing home prefer walking with a robot rather than walking alone [24]. Another study showed that older adults actively participated in robot-assisted cognitive therapy and physiotherapy sessions, and a trend toward improved neuropsychiatric symptoms, reduced apathy, and higher quality of life was observed [25]. Although these studies [12,13,20-25] provide first insights into the acceptance of humanlike robots assisting in group activities of older adults, we identified only 1 study that systematically developed and used an observation system for examining the engagement of groups of older adults during activity sessions assisted by a humanlike social robot [13]. Even though this observation system indicates that systematic observation is a fruitful methodological approach in this research context, it does not fully capture the psychological constructs of engagement and mood that are at the center of our pilot study and that are usually captured using self-report surveys.

### Observation of Engagement and Mood During a Robot-Assisted Group Activity

#### Engagement

In the context of group activities providing physical and cognitive stimulation, engagement in exercises is crucial to generate the intended effects [26]. According to Perugia and colleagues [27], engagement is defined as “the psychological state of well-being, enjoyment, and active involvement that is triggered by meaningful activities and causes people with dementia to be absorbed by the activity, more energetic and in a more positive mood.” Studies with children provide evidence that children are just as willing to engage in robot-guided exercises as when a human demonstrates the exercise [28].

#### Mood

The mood (ie, positive or negative affect) in the group during a robot-assisted activity is of interest to determine if older adults are enjoying themselves in the process, which is relevant to ensure participation beyond curiosity and to assess whether the intended positive effects of such robot-assisted group activities are actually attained. Assessing mood separately from engagement was important also because the stimulus for activities of older adults in the nursing home is a key factor in whether engagement occurs [29]. The general experience is that humanlike social robots, with their ability to express emotions, tend to evoke a notably positive affect. However, the counter hypothesis to this would be that older adults simply want to be polite and participate because something new is happening in the nursing home, without they actually experiencing the positive affect when interacting with the humanlike social robot.

#### Assessment of Engagement and Mood

The assessment of engagement and mood during a robot-assisted group activity has not been researched much [27], and collecting data with older adults in terms of reliable outcomes presents a challenge [7]. Several observational studies have provided inspiration for the design of the observation tool used in this study [30-33]. One important observational instrument is the Observational Measurement of Engagement [34], and its further development can be used to gain a broad understanding of engagement in the context of telepresence robots and companion robots [20]. Although this instrument was not directly suitable to measure the predefined behavior of older adults (eg, mimic an exercise) at the group level, it informed our methodological decisions and developments.

## Methods

### Study Design

We considered this as a pilot study because we developed and tested the applicability of a systematic observation system for rating participants’ engagement and mood during robot-assisted group activity sessions for older adults in nursing homes.

### Recruitment Strategy

A pool of about 200 nursing homes in the German-speaking part of Switzerland were contacted by telephone and invited to participate in this study. Four nursing homes expressed their interest, and they were selected to participate in this observational field study. All participating nursing homes provide various services for leisure activities and physical and cognitive stimulation. They offer accommodation and care to 50-160 residents and provide specialized dementia care. As part of this study, the management of each nursing home agreed to co-organize a robot-assisted group activity together with the research team and made nursing staff available to accompany residents to the session. Residents of the participating nursing homes were informed about the robot-assisted group activity and the study procedure, and they were invited to participate on a voluntary basis.

### Materials

The robot used for the robot-assisted group activity was the NAO robot from SoftBanks Robotics [35]. We used the software of Avatarion [36] developed by Smart Companion [37]. The software was developed in collaboration with experts for leisure activities and physical and cognitive stimulation for older adults, specifically for robot-assisted leisure activities during their care. In this study, we used 3 software modules that support common elements of group activities for older adults: singing, storytelling, and gymnastics.

1. Singing: In the first module, the robot animates the residents to sing along with him or her by using friendly verbal communication and gestures. All songs implemented in this module are well-known Swiss songs that are popular with the older generation. The robot sings the songs with a human voice, and the singing is accompanied with suitable gestures. For songs with more complex lyrics, the residents received handouts of the lyrics.
2. Storytelling: In the second module, the robot tells a story to the residents. The stories are designed to include biographical aspects. All stories implemented in this module are short and contain elements to imitate movements.
3. Gymnastics: In the third module, the robot guides the residents to imitate physical exercises by using friendly verbal communication and gestures. The physical exercises are designed for older adults. For example, the robot shows how to stretch the arms or move the fingers.

**Figure 1:**
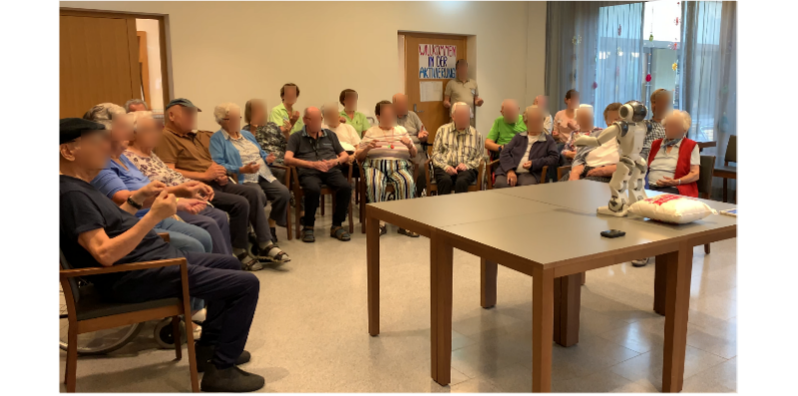
An illustration of a robot-assisted group activity in a nursing home.

**Figure 2:**
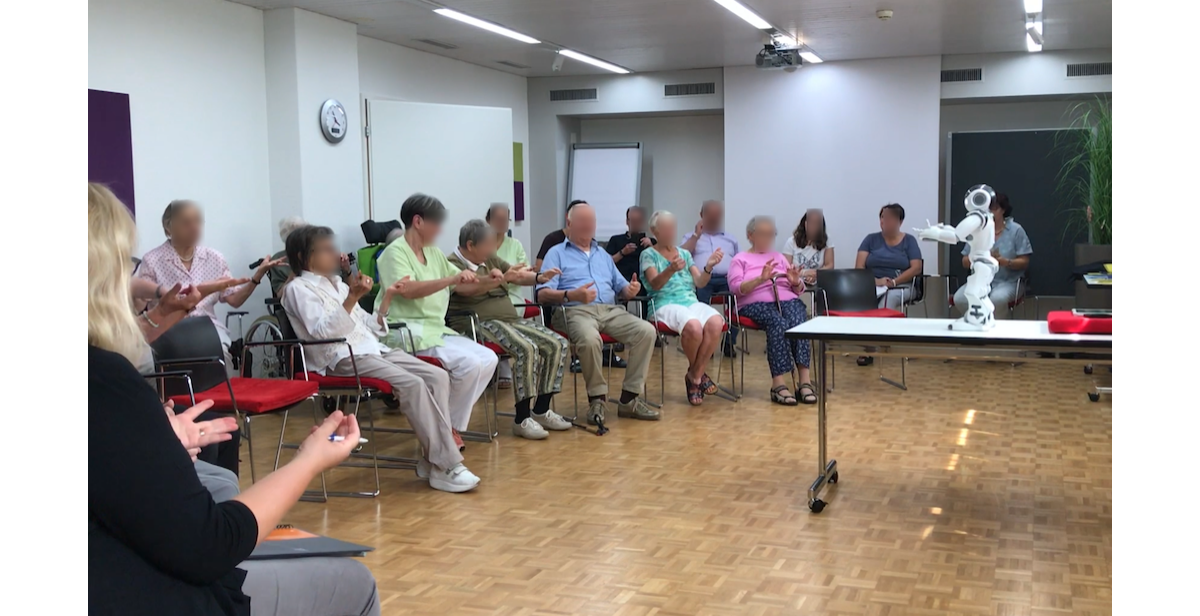
An illustration of a robot-assisted group activity in another nursing home.

Figures 1-2 illustrate the robot-assisted physical exercise sessions in 2 different nursing homes. Photos were taken during 2 robot-assisted group activity sessions in 2 different nursing homes. The pictures show the NAO robot demonstrating movements with its hands and residents participating in this physical exercise by imitating the robot’s movements.

### Ethical Considerations

According to Swiss law this study did not require formal ethics approval and was thus exempt from formal ethics review. For more information please see the corresponding section of the Swiss Human Research Act. The participating nursing homes consented to this study and informed the residents in advance about the robot-assisted activity sessions. Participation in the robot-assisted activity sessions was voluntary. Consent was obtained for using the anonymized photographs in this paper.

### Study Procedure and Data Collection

Robot-assisted group activity sessions were offered in the participating nursing homes in July and August 2019. Chairs and free spaces for wheelchair users were arranged in a way that allowed the participants to see the NAO6 robot that was placed on a table. All robot-assisted group activity sessions took place 1 hour before lunch. Participating residents arrived independently. During the session, 2-5 health care professionals were available in the room for the general support of the residents. All group activity sessions in this study were conducted by research team members and lasted 1 hour, with the actual robot-assisted group activity taking about 30 minutes.

The activity session was structured in 3 parts. First, a representative of the research team welcomed the residents, explained the procedure of the session, reminded them that participation was voluntary, informed them about data protection issues, asked for their approval regarding video recording, and introduced the persons involved. Second, a technical expert from the Smart Companion team started the robot program. The participants first performed a gymnastics exercise, then sang a song together with the robot, and toward the end of the session, they listened to a story. The research team did not interact with the participating residents during these sessions. Third, an additional exercise was conducted by the robot to get the residents in the mood for lunch. This exercise was not recorded and was not part of this study, as it did not aim at their physical and cognitive stimulation. At the end of the activity session, the robot wished the residents bon appétit and said goodbye. Finally, the research team also said goodbye and thanked the residents for their participation.

To systematically analyze residents’ engagement and mood in group activities with the robot, sessions were recorded on video. Video recording has the advantage that, for example, behavior can be observed unobtrusively and participants do not have to be bothered afterwards, as they would be when using interviews. Further, for a high number of residents in nursing homes, other forms of data collection such as surveys present an inaccurate form of assessment, since retrieval, reporting, and ranking of relevant information may be compromised. Therefore, almost all assessment techniques for people with dementia rely on behavior observation [27]. Video recording was done in a way such that residents should not be disturbed, and the Hawthorne effect could be reduced [38]. Hence, short video clips of all 3 exercises were filmed as discreetly as possible. The video clips lasted between 30 seconds and 3 minutes and were distributed across the whole duration of the 3 exercises. The time of the start of the clip in the exercise was chosen randomly. For practical reasons, video clips were recorded with a smartphone camera. For ethical reasons, we collected no personal data such as the age of participants as well as the presence and severity of dementia symptoms. The videos only show the number of participants during each session.

### Measures

For a structured observation of engagement and mood during a robot-assisted group activity, an observation system was developed. The observation system builds on existing observation systems for engagement and mood of individuals but was adapted for direct observation in a group setting. For example, in studies of children’s engagement during one-on-one interactions with robots [30,31], the variables used to measure engagement were direction of gaze, facial expressions, responses, or gestures. Another study related to children with autism spectrum disorders interacting with social robots [32] used measures of engagement based on nonverbal behavior focusing on social and antisocial behaviors. Another system used for older adults observed in a session with a social robot includes measures of engagement and mood targeted to a setting with small groups and a facilitator. Engagement was measured by someone leaning toward the collaborator, and mood was assessed by movements that were accompanied by a positive or negative affect [27]. Further, we analyzed the Observational Measurement of Engagement. This tool is based on a self-identity questionnaire and the 3 dimensions of observational measurements, namely, duration, attention, and attitude. This instrument did not meet all our needs, as we had a predefined duration of an interaction, and attitude was not the focus of our study. However, attention was in our interest and was included in our observation instrument. Another study measured affect and social interaction during a game [33]. Positive affect included smiling and clapping, and negative affect included sadness and anger [33]. Both studies show the relevance of gaze direction for capturing engagement and of observable behaviors for capturing positive and negative affect.

### Engagement

Extending the previous research, we aimed to assess participants’ engagement in robot-assisted group activities. We adapted an established rating system that has been used for the observation of students’ attention in class [39]. This observation system captures 3 aspects of engagement: (1) direction of gaze (looks toward the teaching center vs looks elsewhere), (2) posture and body expression (oriented toward the teaching center and alert vs averted or flaccid), and (3) activity (performs the activity necessary for the task vs does something else on the side). Since we analyzed groups of nursing home residents and were not interested in individual differences, we assessed engagement of the group as a whole. To do so, we created a 5-point rating scale reflecting the degree of engagement in the group. For example, in the original systematic behavior observation instrument [39], sequences were rated whether a child looks toward the teaching center. We have modified the formulation from “none” of the participants looks (score 1) to “all” participants look to the center of the robot-assisted activity (score 5), and this 5-level rating scale aimed to assess engagement from very low to very high (see Table 1). Therefore, we distributed the number of people who showed the behavior depending on group size on the 5-level scale.

**Table 1:** Description of the rating system for engagement at the group level.

Rating	Engagement	Direction of gaze	Posture and body expression	Activity
1	Very low	None of the participants look to the center of the robot-assisted group activity (looking elsewhere)	None of the participants turned toward the center of the robot-assisted group activity but turned away and were flaccid	None of the participants perform the activity necessary for the task, for example, performing movements, singing, or listening to the story told by the robot (doing something else on the side)
2	Low	Most participants do not look to the center of the robot-assisted group activity	Most of the participants are not turned toward the center of robot-assisted group activity but turned away and were flaccid	Most participants do not perform the activity necessary for the task, for example, performing movements, singing, or listening to the story told by the robot (doing something else on the side)
3	Medium	Some participants look to the center of the robot-assisted group activity	Some participants are turned toward the center of robot-assisted group activity and their body expression is alert (vs turned away and flaccid)	Some participants perform the activity necessary for the task, for example, performing movements, singing, and listening to the story told by the robot (vs doing something else on the side)
4	High	Most participants look to the center of the robot-assisted activity session	Most participants are turned toward the center of robot-assisted group activity and their body expression is alert (vs turned away and flaccid)	Most participants perform the activity necessary for the task, for example, performing movements, singing, and listening to the story told by the robot (vs doing something else on the side)
5	Very high	All participants look to the center of robot-assisted activity session	All participants are turned toward the center of robot-assisted group activity and their body expression is alert (vs turned away and flaccid)	All participants perform the activity necessary for the task, for example, performing movements, singing, and listening to the story told by the robot (vs doing something else on the side).

### Mood

To capture participants’ mood during the robot-assisted activities, we developed an observational rating scale based on the German version of the Positive and Negative Affect Schedule (PANAS) [40]. The PANAS is frequently used in studies in which human mood states are of interest. The questionnaire consists of 20 adjectives describing different feelings with 10 adjectives capturing positive affect and the other 10 capturing negative affect. The items of the original PANAS are shown in Textbox 1 [25]. This survey instrument was chosen because it contains a set of mood variables that describe mood with positive and negative affects with several adjectives that we assumed were observable by a rater. Based on findings by Reisenzein and colleagues [41] that emotions can be detected by observers using a variety of cues (eg, facial expressions, verbal expressions, physical expressions), we transformed the survey instrument PANAS into an observational rating scale for mood at the group level.

**Adjectives of the Positive and Negative Affect Schedule.**

**Positive affect**

- Attentive
- Active
- Alert
- Excited
- Enthusiastic
- Determined
- Inspired
- Proud
- Interested
- Strong (this mood could not be observed reliably in our study)

**Negative affect**

- Hostile
- Irritable
- Ashamed
- Guilty (this mood could not be observed reliably in our study)
- Distressed
- Upset
- Scared
- Afraid
- Jittery
- Nervous

The original 5-level response scale contains the gradations “very slightly or not at all,” “a little,” “moderately,” “quite a bit,” and “extremely.” Again, because we were interested in the mood at the group level, we adapted the rating scale to reflect observable indicators of mood in the group, and a 5-point rating scale from “very low” to “very high” was used. For example, “very low” signified none of the participants were attentive in the robot-assisted group activity, and “very high” signified all participants were attentive in the robot-assisted group activity (see Table 2). To make an objective assessment of group mood during the robot-assisted group activity, sequences from the observation were rated in relation to each adjective from the PANAS. The description of the 5-point rating scale of mood according to the PANAS is shown in Table 2. We considered the observation at group level to be particularly relevant for capturing mood in the group so that situational factors that are an important component in the observation of mental states [41] could be included.

**Table 2:** Description of the 5-point rating scale of mood according to the Positive and Negative Affect Schedule [35] for a robot-assisted group activity.

Rating	Extent of the perceived states for the measurement of mood in the group activity	Description
1	Very low	None of the participants are __^a^ in the robot-assisted group activity.
2	Low	Most participants at the robot-assisted group activity are not __.
3	Medium	Some participants are __ in the robot-assisted group activity.
4	High	Most participants are __ in the robot-assisted group activity.
5	Very high	All participants at the robot-assisted group activity are __.

^a^The rating system was used for every adjective of the Positive and Negative Affect Schedule.

### Coding of Video Recordings

Video clips were rated independently by 2 trained observers (rater 1 and rater 2). Rater 1 was present during all robot-assisted group activities and rater 2 during two randomly selected sessions. Both raters were trained in the observation of nonverbal communication and body language for assessing the items for all 3 aspects of engagement (ie, direction of gaze, posture and body expression, activity) and for mood (ie, positive and negative affect). Clearly visible signs of dementia and severe physical limitations of the residents had to be considered, and the rating of engagement had to be adjusted to the residents’ possibilities of participation (eg, physical limitations). However, no individual was excluded from the analysis, as all ratings were performed at the group level. Each observer rated the group as a whole in every video clip by assessing whether none of the participants, some of the participants, most of the participants, or all of the participants exhibited a particular behavior indicating engagement (eg, direction of gaze, posture and body expression, activity) or positive or negative affect (eg, attentive, scared). To provide specific and context-sensitive anchors for the ratings, we counted the number of participants and distributed them proportionally across the 5-point scale. Thus, it depended on the actual group size what most and some participants meant. During the initial trial, it became apparent that all variables of engagement could easily be observed and rated by both raters. However, for rating the perceived mood in the group of study participants according to PANAS, additional coding rules had to be defined. The items “strong” and “guilty” were difficult to observe and hard to differentiate from 2 other items (eg, proud, ashamed) and thus not considered in the analysis. Each video clip was rated independently by the 2 raters to allow for reliability assessment.

### Data Analysis

#### Interrater Agreement

To evaluate the agreement between 2 raters, we calculated the intraclass correlation coefficient (ICC) with the SPSS statistics software (version 26; IBM Corp). An ICC higher than 0.61 was considered substantial, and ICC higher than 0.81 was considered an almost perfect agreement [42].

#### Video Analysis

The number and gender of participants who attended each robot-assisted group activity session was extracted from the videos and presented descriptively. For the analysis of engagement, the mean values, standard deviation, and medians of the aspects direction of gaze, posture and body expression, and activity as well as the overall mean value, standard deviation, and median of engagement were calculated from the observers’ ratings. We also calculated the mean value, standard deviation, and median for each item and the positive and negative affect dimensions from the PANAS for each robot-assisted group activity session.

## Results

### Videos and Study Participants

Of the 34 video clips recorded during 5 robot-assisted group activities, 3 videos had to be excluded because not all participants were visible or the video was too short to be rated. Thus, we finally included 31 video clips. In the 4 participating nursing homes, 85 older residents participated in 5 robot-assisted group activity sessions. Participant characteristics are provided in Table 3.

**Table 3:** Characteristics of the nursing homes and attendance in the activity sessions.^a^

Participating long-term care facilities	Residential spaces (n)	Residents attending the group activity (N=85)
1	160	15
2	140	20
2	140	16
3	82	18
4	48	16

^a^In nursing home 2, we conducted 2 independent robot-assisted group activity sessions.

### Interrater Agreement

Agreement between the 2 raters was high for engagement and positive and negative affect. Engagement had an ICC score of 0.83 (95% CI 0.65-0.92). Negative affect reached an ICC of 0.84 (95% CI 0.67-0.93), and positive affect had an ICC of 0.90 (95% CI 0.79-0.96). Individual items, specifically adjectives that belonged to negative affect, received a rather weak ICC. These include the items “ashamed” (ICC 0.37, 95% CI –0.32 to 0.70) and “afraid” (ICC 0.39, 95% CI –1.07 to 0.52).

### Engagement

As Table 2 demonstrates, the results show that the engagement of the participants in the robot-assisted group activity was high (mean 4.19, SD 0.47; median 4.0). The direction of gaze was measured as almost very high (mean 4.65, SD 0.49; median 5.0); posture and body expression (mean 4.03, SD 0.71; median 4.0) and activity (mean 3.90, SD 0.65; median 4.0) were also rated as high.

### Mood

Overall, no negative affect could be observed (mean 1.13, SD 0.20; median 1.0). The mean value of positive affect was 3.22 (SD 0.55; median 3.2), which indicates the observer perceived a good mood during the sessions. Adjectives of the positive affect such as interested (mean 4.13, SD 0.56; median 4.0), alert (mean 4.39, SD 0.67; median 4.0), inspired (mean 3.87, SD 0.96; median 4.0), attentive (mean 4.19, SD 1.05; median 4.0), and active (mean 4.16, SD 0.64; median 4.0) received high ratings around the value 4, while enthusiastic (mean 2.42, SD 1.03; median 2.0), proud (mean 1.23, SD 0.43; median 1.0), and determined (mean 1.94, SD 0.77; median 2.0) were observed to be very low or low within the group of participants. Table 4 shows the detailed results.

**Table 4:** Interrater agreement as well as the mean (SD) and median values for the study variables engagement and mood (ie, positive and negative affect) observed during robot-assisted group activities.^a^

		Interrater agreement (intraclass correlation coefficient)	Mean (SD)	Median
**Engagement**		0.831	4.19 (0.47)	4.0
	Direction of gaze	0.661	4.65 (0.49)	5.0
	Posture and body expression	0.883	403 (0.71)	4.0
	Activity	0.811	3.90 (0.65)	4.0
**Positive affect**		0.902	3.22 (0.55)	3.2
	Interested	0.842	4.13 (0.56)	4.0
	Excited	0.825	2.61 (0.92)	3.0
	Enthusiastic	0.840	2.42 (1.03)	2.0
	Proud	0.680	1.23 (0.43)	1.0
	Alert	0.750	4.39 (0.67)	4.0
	Inspired	0.884	3.87 (0.96)	4.0
	Attentive	0.901	4.19 (1.05)	4.0
	Determined	0.766	1.94 (0.77)	2.0
	Active	0.722	4.16 (0.64)	4.0
**Negative affect**		0.840	1.13 (0.20)	1.1
	Distressed	0.842	1.29 (0.59)	1.0
	Upset	0.768	1.16 (0.52)	1.0
	Scared	0.659	1.06 (0.43)	1.0
	Hostile	0.491	1.10 (0.48)	1.0
	Irritable	0.649	1.06 (0.25)	1.0
	Ashamed	0.365	1.16 (0.37)	1.0
	Nervous	0.804	1.29 (0.53)	1.0
	Jittery	0.665	1.23 (0.43)	1.0
	Afraid	0.390	1.13 (0.51)	1.0

^a^The items “strong” and “guilty” were not analyzed.

### Additional Observations

Although we did not collect this information systematically, we observed that more residents participated in the robot-assisted activity sessions than expected by the nursing home staff and the research team. The different types of robot-assisted exercises (ie, singing, storytelling, gymnastics) promoted a variety of cognitive and physical stimulations as would a human instructor. Further, when watching the video recordings, we noted that the nursing home staff took time to assist and support participants individually during the robot-assisted activity session. Conversations took place between the residents and the nursing staff, and it seemed that the robot conducting all the instructions allowed more time for personal care.

## Discussion

In robot-assisted group activity sessions for older adults in nursing homes, their engagement and mood (ie, positive affect) can be regarded as preconditions to achieve the intended positive effects of physical and cognitive stimulation. Our observational pilot study in 4 nursing homes shows that residents actively engage in the leisure activities demonstrated and guided by a humanlike social robot. Overall, the engagement of the older adults in gymnastics exercises, singing with the robot, or listening to the robot telling stories was high. Engagement in the group activity was measured using 3 variables: direction of gaze, posture and body expression, and activity that the robot demonstrated. Almost all participants in the robot-assisted activity sessions kept their gaze directed toward the robot, and most had an active alert posture and actively imitated the movements demonstrated by the robot. We observed a positive mood in the groups during the robot-assisted activity sessions. Overall, the items measuring positive affect received high ratings, and the mood in the groups was mainly interested, alert, inspired, and attentive. The results of our study extend and complement existing laboratory studies as well as studies applied in areas other than the nursing home [13] by systematically using observational data to gain a better understanding of the ways in which residents engage in and experience robot-assisted group activities. From a methodological point of view, the participatory observation with video recording provided new insights. The systematic coding of video clips using structured observation systems for both study variables allowed us to reliably show whether participants engage with a positive mood. Further, the observation system developed for this study complements existing instruments for measuring engagement and positive and negative affect by focusing on group level measures and the behavior toward a humanlike social robot in a group activity.

In contrast, other instruments [2,27,34] focus on engagement-related behavior, wherein older adults directly interacted in a one-on-one setting with the robot and not within a group activity. During a group activity, it is common for older adults, especially for those with physical limitations and early signs of dementia, to express behavior less consistently and clearly. By observing at group level, it was possible to assess the engagement and the mood of the individual in the situation and context of the group, and the sometimes subtle cues to emotion could be reliably detected by the trained raters. This is important as the technical recognition system still needs to be greatly improved [36]. The added value of the instrument is that it allows for monitoring engagement and mood in a group of participants with limited self-report capabilities and thus broadens the insights gained with the existing instruments. In combination with the initial findings from other field studies [13,21,23,25,43] specifically studying the fostering of well-being in a nursing home setting, our results show the potential of such activity sessions to make a valuable contribution. For example, a memory study program with a humanlike social robot for older adults for cognitive training in a nursing home showed positive trends [44] and could be adapted for fun group activities.

As limitations, the following aspects should be mentioned. First, participants attended the robot-assisted group activity sessions voluntarily and were generally informed about the content beforehand; so, when they joined the session, they may have had a positive attitude toward robots, which could therefore have introduced a bias toward a more positive affect. Moreover, although the reliability of the observation system could be shown with high ICC values for most items measuring affect, some mood items had to be excluded due to difficulties in distinguishing them through observation during short interactions in pretests and some items still have rather low ICC values (eg, ashamed, afraid). This indicates that negative affect was more difficult to assess, which needs to be reflected critically when interpreting our findings. This result matches the findings of a study that measured emotions of individuals with severe intellectual disabilities where positive emotions were also found to be more observable than negative ones [45]. Thus, the investigation of negative affect while participating in robot-assisted activities might be an interesting focus of future studies. Second, we did not collect data as to whether the participants had mild or severe dementia. Although the analysis at the group level allowed consideration of situational factors and the constraints of the individuals were included by the raters, there may be differences in engagement and mood expression depending on the level of dementia as previous research shows [46]. Third, each robot-assisted activity was only performed once per group. Thus, we were unable to assess sequence effects or analyze which activities are the most engaging or which activities might tire participants more quickly. Moreover, in this study, we did not have the opportunity to study engagement and mood over a long period of time. Future research is needed for this [47]. Thus, novelty effects cannot be excluded. Interestingly, some studies [23] over longer periods of time did not report an attractiveness loss of the robot, but they did mention loss of interest due to usability problems with the robots. The so-called novelty effect [48] theoretically predicts “a decrease in the engagement with a stimulus after its initial novelty has worn off.” Usually, it is seen as a bias that has to be overcome (eg, by repeated interaction with the robot). An experiment with well-controlled repeated interactions showed that perceptions were positively influenced when participants interacted with the robot [48] and reported that a consistently positive interaction was already determined in the first 2 minutes of the conversation with the robot and remained stable over the subsequent sessions. In contrast, perceived threat and discomfort were the dimensions that changed the most during the interactions and decreased until the last session of the experiment [48]. With this in mind, we assume that the engagement and positive mood observed in the initial interaction as in our study are likely to be maintained in a relatively stable manner. Fourth, our pilot study investigates engagement and mood across different exercises within a robot-assisted activity session. In terms of effects on health, a larger study should assess which type of exercise receives the greatest engagement and positive affect, and it is of interest to continuously record the exercises. This allows for a more precise analysis of behavior during exercises, such as fatigue, and facilitates better comparisons of participation in the exercises among themselves. This knowledge would inform future software development and implementation of social robots. Finally, we did not investigate the practicability of the NAO robot for nursing staff and how a robot-assisted group activity can be implemented in a nursing home successfully. Research following a human-centered design approach [49] and an improved understanding of sustainable integration of social robots in leisure activities of older adults during their care are crucial.

Based on the positive findings of our study, questions arise about other application areas for robot-assisted group activities. Group sessions with social robots generate a form of enthusiasm, which is why they may be particularly suitable for group activities with vulnerable groups such as children, older adults, or people with disabilities. For all these potential target groups, interactions need to be designed in a way that results in maximum benefit and does no harm. In the context of the shortage of skilled nursing staff, social robots bring the potential to conduct a leisure group activity where caregivers do not need to be continuously present, thereby enabling older adults to be physically and cognitively engaged with less care effort and with fun. Moreover, if the social robot demonstrates exercises, nursing staff have more time for individual care as well as for personal conversations with the older adults. The literature shows that engagement and mood are prerequisites for health effects to be achieved [26]. Although the generalizability of our results must be established by future research, we found that older adults engage in robot-assisted group activities and that most of them were in a good mood during the session—interested, alert, inspired, and attentive. Therefore, the positive results on engagement and mood provide clear indications that humanlike social robots can improve the cognitive and physical abilities in older adults. Compared to other technologies, robots with their ability to communicate in a humanlike manner have a special property of supporting individuals physically and psychologically. Further development of this new technology of social robots is thus worthwhile in terms of promoting the quality of life of older adults in nursing homes.

## Abbreviations

ICC: intraclass correlation coefficient
PANAS: Positive and Negative Affect Schedule
